# Bullying victimization among adolescents during the early phase of war in Ukraine – A comparative cross‐sectional study in 2016–2017

**DOI:** 10.1111/camh.12770

**Published:** 2025-03-18

**Authors:** Sanju Silwal, Minja Westerlund, Olga Osokina, Susanna Hinkka‐Yli‐Salomäki, Matthew Hodes, Norbert Skokauskas, Andre Sourander

**Affiliations:** ^1^ Research Centre for Child Psychiatry, INVEST Flagship University of Turku Turku Finland; ^2^ Addiction Psychiatry and Medical Psychology Donetsk National Medical University Kramatorsk Ukraine; ^3^ Psychiatry and Physical Rehabilitation Kyiv Medical University Kyiv Ukraine; ^4^ Division of Psychiatry Imperial College London London UK; ^5^ Regional Centre for Child and Youth Mental Health and Child Welfare IPH, Norwegian University of Science and Technology Trondheim Norway; ^6^ Turku University Hospital Turku Finland

**Keywords:** Bullying, adolescence, war, posttraumatic stress disorder, depression

## Abstract

**Background:**

War profoundly impacts adolescent development and may increase the likelihood of aggressive responses when such behavior is perceived as acceptable and accessible. War may, hence, exacerbate a form of interpersonal violence already prevalent among children and adolescents.

**Methods:**

We conducted a comparative cross‐sectional study 2 years after the Russian invasion of Eastern Ukraine in 2014 by comparing the prevalence of bullying victimization among adolescents aged 11–17 years (*N* = 2766) in two administrative regions with different levels of wartime traumatic stressor exposure.

**Results:**

Female adolescents in the war‐affected region were bullied more often compared to those in the non‐affected region [65.3% vs. 56.3%, adjusted Odds Ratio (aOR) = 1.5, 95% CI 1.2–1.9]. For both boys and girls, symptoms of psychopathology were associated with bullying victimization often [girls: depression (aOR = 2.9, 95% CI 2.4–3.4); boys: depression (aOR = 3.3, 95% CI 2.6–4.1) and PTSD (aOR = 1.7, 95% CI 1.4–2.02)]. In the war‐affected region, a dose–response relationship between bullying victimization often and war‐event exposure was observed in both sexes [girls: 1–3 war‐events (aOR = 1.4, 95% CI 0.7–2.6), 4–6 (aOR = 2.4, 95% CI 1.3–4.5) and ≥7 (aOR = 5.5, 95% CI 2.7–11.1); boys: 1–3 (aOR = 1.4, 95% CI 0.7–2.8), 4–6 (aOR = 3.2, 95% CI 1.7–6.3), and ≥7 (aOR = 6.8, 95% CI 3.1–14.8)].

**Conclusions:**

War exposure was associated with bullying victimization, with girls being bullied more often than boys. Bullying victimization was linked to cumulative traumatic stressor exposure in the war‐affected region for both sexes.

**Key Practitioner Message**

**What is known?**

- Based on social learning perspectives, it can be hypothesized that war may increase children's and adolescents' socialization to aggressive responses.

**What is new?**

- Adolescent girls, but not boys, in the war‐affected region were bullied more often compared to those in the non‐war‐affected region.
- For both sexes, psychopathology symptoms were associated with bullying victimization.
- In the war‐affected region, a dose–response relationship between bullying victimization and traumatic stressor exposure was observed in both sexes.
- Compared to witnessing violence, experiencing violence may exert different underlying mechanisms on bullying victimization.

**What is significant for clinical practice?**

- Bullying behavior in adolescents should be examined within a broader macrosystem level, such as in war or armed conflict, and not only at the microsystem level, like in schools or homes.

## Introduction

An increasing number of children and youth are living in the midst of war or armed conflict (UNICEF, 2022). The violence, losses, and broad‐scale devastation of community structures that war entails are strongly linked with psychosocial problems and can exert a profound developmental impact on children and adolescents (Hazer & Gredebäck, 2023). These adverse effects are not only limited to cognitive and emotional development, but war experiences also likely affect social development, including the likelihood of adopting aggressive responses in social situations. According to social learning perspectives, war may increase children's and adolescents' socialization to aggression through observing violence, which can modify or create a belief that violence is an acceptable behavioral strategy to achieve goals (Huesmann, 1988; Perry, Perry, & Rasmussen, 1986; Tolan & Guerra, 1998). In line with the Social Information Processing Theory (Dodge, 1986; Crick & Dodge, 1994; Lemerise & Arsenio, 2000), children's responses to social stimuli result from a stepwise processing of environmental cues in function with their existing biological capabilities and memories of previous experiences. The likelihood of an aggressive response increases when the child or adolescent perceives a threat and when an aggressive response is seen as acceptable and mentally accessible, as is often observed during times of armed conflict (Keresteš, 2006). It could be hypothesized that war exacerbates bullying, which is a particular form of interpersonal violence that is already relatively widespread among children and adolescents.

Bullying is characterized by repetitive aggressive behavior taking place within an imbalance of power relationship that causes harm or distress to the victim (Gladden, Vivolo‐Kantor, Hamburger, & Lumpkin, 2014) and can manifest, for example, as direct‐physical, direct‐verbal, indirect‐relational, or cyberbullying. Bullying is generally considered a subcategory of aggressive behavior (Solberg, Olweus, & Endresen, 2007). The recent World Health Organization's Health Behavior in School‐aged Children (Cosma, Molcho, & Pickett, 2024) survey in 44 countries in Europe, central Asia, and Canada showed that 6% of adolescents reported bullying others and 11% reported being bullied, prevalences that reflect earlier reports (Olweus, 1997). Previous literature has indicated possible higher accuracy of self‐reports on bullying victimization than of self‐reports on bullying perpetration (Branson & Cornell, 2009), likely partly due to social desirability bias. Further, a meta‐analysis indicated that perpetration and victimization overlap strongly (Walters, 2021); however, there are some differing results (Solberg et al., 2007).

Previous literature about the proposed relationship between armed conflict and increased bullying is limited, showing inconsistent results. A study conducted in Colombia among 53,316 fifth and ninth graders showed that exposure to armed conflict predicted bullying for fifth graders; however, with great variation at the school level, and mixed results regarding socioeconomic factors (Chaux, Molano, & Podlesky, 2009). Beliefs supportive of aggression were associated with bullying (Chaux et al., 2009), aligning with the Social Information Processing Theory (On the other hand, a study conducted in postwar Bosnia and Herzegovina among 484 fourth‐to eighth‐grade students did not find differences in bullying rates between adolescents living in war‐exposed and unexposed areas (Obrdalj & Rumboldt, 2008). Expanding beyond the realm of war, Schwartz and Proctor (2000) studied the association between exposure to community violence and peer rejection, bullying by peers, and aggression. They found that community violence (such as killings, stabbings, physical assaults, threats) was associated with social maladjustment regardless of sex and via different mechanisms; witnessing community violence was linked to aggression, proposedly through social learning. Violent victimization, again, was associated with peer rejection, bullying by peers, and aggression, mediated via emotion regulation difficulties. A study conducted in Ukraine 6 years after the Russian invasion in 2014 examined bullying perpetration among 2763 adolescents aged 10–17 years living in proximity to the war zone. The study showed that bullying perpetration was significantly associated with more symptoms of conduct disorder and low parental involvement (Burlaka et al., 2025).

Additional studies have assessed the association between war exposure and other types of aggressive, socially maladjusted behavior in children and adolescents. Keresteš (2006) studied Croatian school children in two towns with differing levels of war exposure. Greater exposure to wartime traumatic stressors was linked with more self‐reported aggression and with teacher‐reported lower prosocial and somewhat increased aggressive behavior. Gender did not moderate the relationship. Finally, in a context of active conflict, Qouta, Punamäki, Miller, and El‐Sarraj (2008) studied Palestinian children living in the Gaza strip exposed to different degrees of military violence and found that military violence exposure was associated with parent‐reported aggressive behavior in the children. The association between military violence exposure and aggression was not dependent on gender.

In 2014, Russian forces invaded and occupied the Crimean peninsula and part of the Donetsk and Lugansk regions of Eastern Ukraine (Bowen, 2019). The present study is the first large epidemiological study that was conducted among adolescents in 2016–2017 from two areas differently affected by war. In our previous study, we reported that adolescents in the war‐affected region were more likely to experience war trauma and an increased risk for posttraumatic stress disorder (PTSD), depression, and anxiety (Osokina et al., 2023). In the present study, we compared the prevalence of bullying victimization among adolescents in the Donetsk region, which was directly exposed to war during the early phase of the war, and the Kirovograd region, which was not affected directly by war. Based on previous literature, we hypothesized that greater exposure to wartime traumatic stressors would be associated with increased bullying rates. We expected that experiencing war‐related violence would be associated with bullying victimization, regardless of sex, and that this relationship would be mediated by PTSD and depression symptoms; conditions where emotional dysregulation plays a central role. Socioeconomic factors were studied exploratively.

## Methods

### Participants

This cross‐sectional school‐based study was carried out 2 years after the 2014 Russian invasion of areas of Eastern Ukraine. Participants were adolescents from government‐funded schools in the Donetsk region, which had experienced war since 2014, and the Kirovograd region in central Ukraine, a peaceful region at that time. Convenient sampling was used to select cities and schools for the study. Kramatorsk, Sloviansk, and Druzhkovka, located in eastern Ukraine, were temporarily captured by Russian forces in 2014 and later liberated by the Ukrainian army. After the occupation of Donetsk, many families moved from Donetsk to these liberated cities. In 2016–17, Kramatorsk, Sloviansk, and Druzhkovka were the nearest Ukrainian‐controlled cities to the frontline and had directly experienced the violence of war. To compare the mental health consequences of war on adolescents from war‐affected and non‐war‐affected regions, we selected Kirovograd, located 500 km from Donetsk in central Ukraine, which was not directly affected by the war. Most schools contacted in both regions agreed to participate in the study. From September 2016 to January 2017, researchers selected 15 schools: nine from Kramatorsk, Sloviansk, and Druzhkovka in Donetsk and six from Kropyvnytskyi and Alexandria in the Kirovograd region. Teachers explained the study, and informed consent was distributed to parents. Those with parental consent were enrolled.

**Figure 1:**
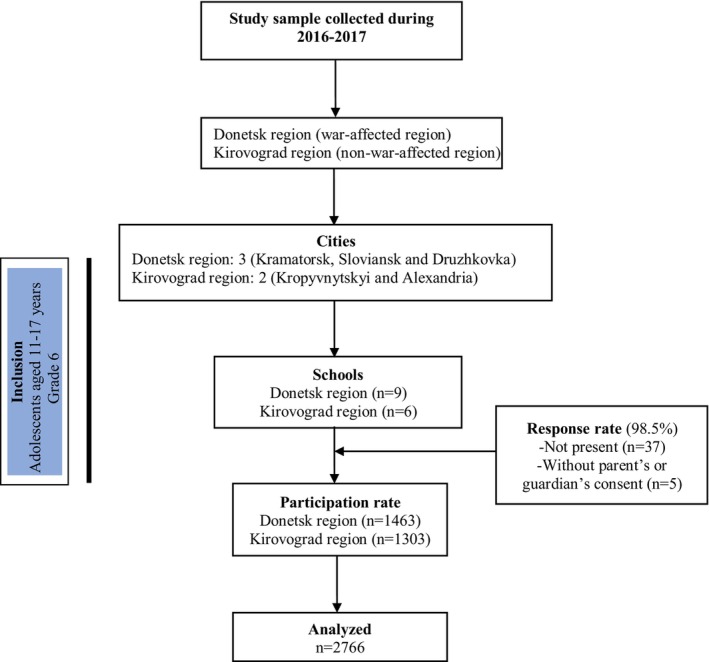
STROBE flowchart. STROBE, Strengthening the Reporting of Observational Studies in Epidemiology

The target population comprised 2803 adolescents aged 11–17 years. Of those, 37 (11: Donetsk; 26: Kirovograd) were absent on the day of data collection, and five parents refused to give consent (2: Donetsk; 3: Kirovograd) leaving a final sample of 2766. Response rates were 98.5% in the Donetsk and 97.2% in the Kirovograd regions. Figure 1 shows the flowchart of the study. The assessment, conducted in classrooms, took about 45 min, with completed questionnaires sealed and submitted to researchers or teachers. This study followed STROBE guidelines (von Elm et al., 2007), and the checklist is included in the Appendix.

### Measures

All psychopathology measures were translated from English into Ukrainian and Russian and back‐translated to English for accuracy. Ethical approval was obtained from the ethical committee for the medical research ethics of Donetsk National Medical University, Ukraine on May 11, 2016, number 16.

#### Demographic characteristics

Demographic information included sex (girl or boy), age (<13 and ≥13 years), region of residence (Donetsk region or Kirovograd region), parental employment status (employed or unemployed), and family structure (living with two biological parents or other). ‘Other’ included living with one parent, a foster family, grandparents, or an adopted family.

#### Bullying victimization

Adolescents reported their bullying experiences. Bullying in the questionnaire was defined as: ‘A student is being bullied when he or she is exposed repeatedly over time to negative and hurtful actions by one student or group of students. Bullying can be intermittent or continuous and it is difficult to defend themselves. Bullying can be verbal (e.g. calling names, threats), physical (e.g. hitting or pushing) or psychological (e.g. spreading rumors, exclusion)’. Two questions assessed bullying: (i) how often you have been bullied in school in the past six months? and (ii) How often you have been bullied away from school in the past six months? Responses were categorized into four: not at all, less than once a week, more than once a week and most days. For this study, ‘less than once a week’ was categorized as ‘sometimes' while ‘more than once a week’ and ‘most days' combined as ‘often’. Thus, final categories were: ‘never’, ‘sometimes’ and ‘often’. This measure has been previously used in another study among adolescents (Chudal et al., 2022).

#### Wartime traumatic stressor exposure

Exposure to wartime traumatic stressors was assessed using original questions developed by Ukrainian child and adolescent psychiatrists and psychologists working with adolescents exposed to war. The questionnaire was designed based on stressful experiences that increase the risk for depression and other psychological problems (Macksoud, 1993), the Harvard Trauma Questionnaire (Mollica et al., 1992), and specific wartime traumatic stressors from the local military conflict in eastern Ukraine. The list of wartime traumatic stressors was reviewed by a small sample of adolescents, and necessary modifications were made based on their feedback. The questionnaire comprised 12 items about different types of wartime traumatic stressors, with yes/no responses. The exposures were summed with scores ranging from 0 to 12. We were interested in studying the associations between levels of wartime traumatic stressor exposure and victimization; thus, we categorized the number of exposures into four groups: no wartime traumatic stressor exposure, 1–3 exposures, 4–6 exposures, or seven or more different types of exposures.

#### Posttraumatic stress symptoms

Symptoms of PTSD were assessed with the Harvard Trauma Questionnaire (HTQ), which has been widely used in adults and adolescents (Mollica et al., 1992). The first 16 items correspond to the PTSD criteria in the Diagnostic and Statistical Manual of Mental Disorders, Fourth Edition (DSM‐IV). The respondent rates the frequency of symptoms experienced during the last week, rated on a scale that ranges from zero (*not at all*) to four (*extremely*).

#### Depression symptoms

Symptoms of depression were assessed using the nine‐item Patient Health Questionnaire (PHQ‐9), a self‐administered tool that has been widely used among adults and adolescents (Kroenke, Spitzer, & Williams, 2001). The respondent rates how frequently they experienced symptoms over the last 2 weeks on a 4‐point Likert scale, ranging from 0 to 3.

### Statistical analyses

Descriptive analysis was used to summarize demographic characteristics. Means and standard deviations were calculated for continuous variables, and frequencies and percentages were calculated for categorical variables. The chi‐square test was used to examine differences in categorical variables. Multinomial logistic regressions were conducted to examine the associations between bullying victimization and explanatory variables in a single model. Odds ratios (OR) together with 95% confidence intervals (CI) were used to estimate the strength of the associations. Statistical significance was based on a two‐sided *p* < .05. Additionally, a two‐way interaction effect was run between region and demographic factors: sex, age, parental employment status, family structure, and psychopathology. All statistical analyses were conducted using SPSS statistical software, version 29.0 (IBM Corp, Armonk, NY, USA). Due to limitations placed by the ethical approval for the study, the data cannot be publicly shared.

## Results

Table 1 presents the characteristics of the sample by regions. There were no significant differences between regions in terms of sex, age, mother's employment status, and family structure, except for father's employment status. More fathers in the war‐affected region were unemployed (*p* < .001). There were significant differences in PTSD and depression symptom scores between regions (*p* < .001).

**Table 1:** Demographics characteristics of the sample

	Donetsk region (war‐affected)	Kirovograd region (non‐war‐affected)	*p*‐Value
	(*n* = 1463)	(*n* = 1303)	
Sex			.09
Male	693 (47.4)	660 (50.7)	
Female	770 (52.6)	643 (49.3)	
Age			.54
<13 years	527 (36.0)	484 (37.1)	
≥13	936 (64.0)	819 (62.9)	
Mother's employment status			.98
Employed	1139 (77.9)	1015 (77.9)	
Unemployed	324 (22.2)	288 (22.1)	
Father's employment status^a^			<.001
Employed	1220 (83.4)	1238 (95.0)	
Unemployed	242 (16.6)	65 (5.0)	
Family structure			.73
Biological parents	916 (62.6)	824 (63.2)	
Others^b^	547 (37.4)	479 (36.8)	
Wartime traumatic stressor exposure			<.001
No exposure	224 (15.3)	1078 (82.7)	
1–3	209 (16.0)	209 (16.06)	
4–6	416 (28.4)	4 (0.3)	
≥7	190 (13.0)	12 (0.9)	

	*M* (*SD*)	*M* (*SD*)	
PTSD symptom scores	1.5 (0.5)	1.4 (0.4)	<.001
Depression symptom scores	4.3 (5.4)	3.6 (4.1)	<.001

^a^ Missing 1.

^b^ Living with one parent, a foster family, grandparents or an adopted family.

**Figure 2:**
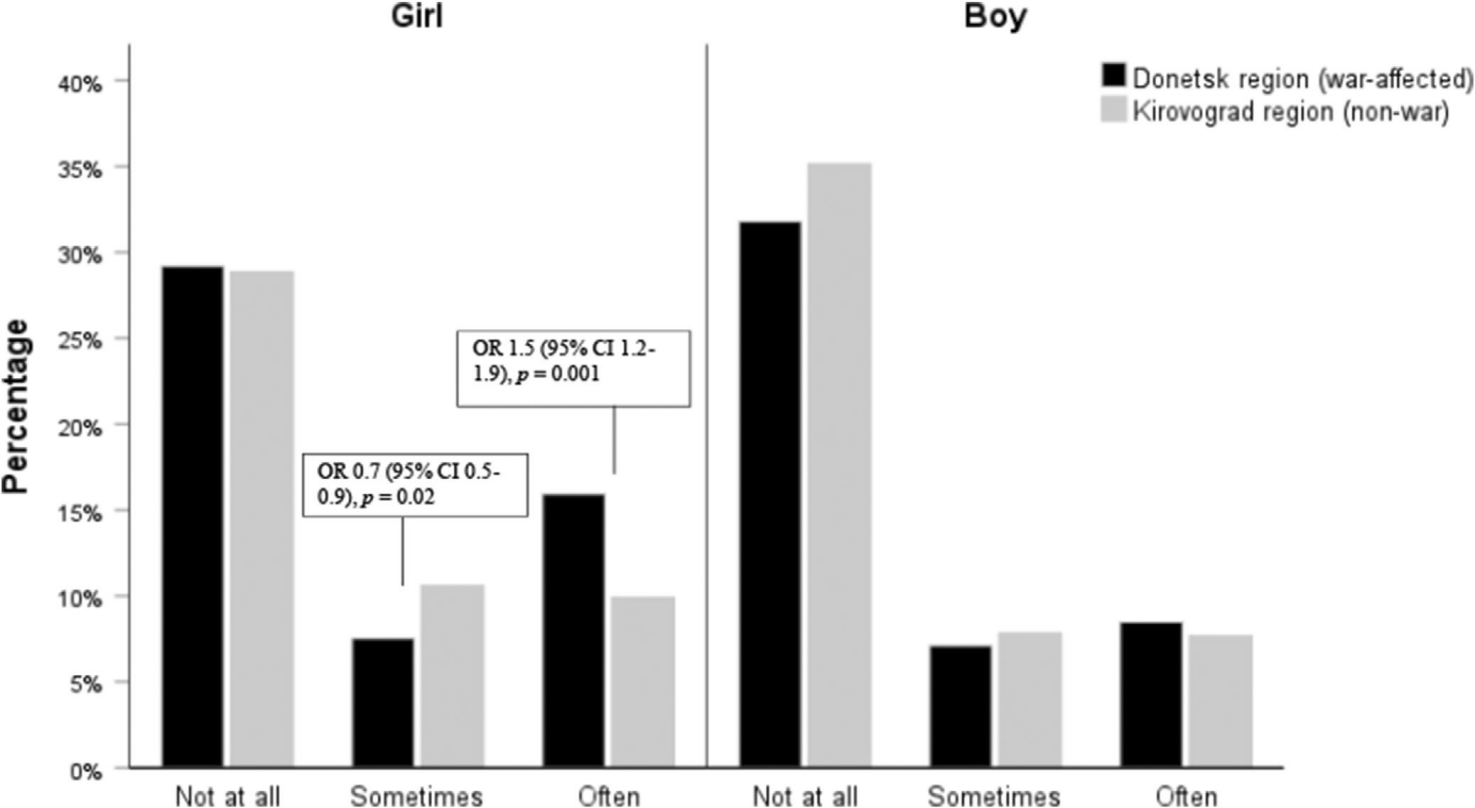
Bullying victimization between war‐affected and non‐war regions and by sex. Results from multinomial logistic regression

Figure 2 shows bullying victimization in the war‐affected and non‐war‐affected regions by sex. There was a significant city by sex interaction (*p* < .001); thus, results were reported separately for boys and girls. Multinomial logistic regression was used to examine their associations. Girls in the war‐affected region reported being victimized more often compared to those in the non‐war‐affected region (65.3% vs. 56.3%, aOR = 1.5, 95% CI [1.2–1.9]), see Table S1. About 65 out of 100 girls in the war‐affected region reported being victimized often. No significant differences were observed among boys between war‐affected and non‐war‐affected regions. In the war‐affected region, there were significant sex differences in bullying victimization, with girls reporting being victimized more often than boys (65.3% vs. 34.7%, aOR = 2.04, 95% CI [1.6–2.6]). Similar results were observed in the non‐war‐affected region, with a higher prevalence of victimization often and sometimes observed in girls than boys (56.3% vs. 43.7%, aOR = 1.6, 95% CI [1.2–2.1]; 57.5% vs. 42.5%, aOR = 1.7, 95% CI [1.2–2.2]).

Table 2 shows bullying victimization by sex. In both sexes, older age ≥13 years were bullied often (girls:72.4%, boys: 62.1%), were from war‐affected regions (64.4% vs. 55.4%). The mean scores of PTSD and depression were statistically higher in girls than boys; PTSD scores were (1.5, 0.5 vs. 1.4. 0.4; *p* < .001); depression scores (4.9, 5.4 vs. 2.9, 3.9, *p* < .001). Table 3 shows the association between bullying victimization and independent variables. The reference category was bullying victimization never. Adolescent girls with depression reported being victimized often (aOR = 2.9, 95% CI [2.4–3.4]) and sometimes (aOR = 1.4, 95% CI [1.2–1.6]). Those with PTSD reported being victimized sometimes (aOR = 1.7, 95% CI [1.5–2.1]). Adolescent boys with depression and PTSD reported being victimized often (aOR = 3.3, 95% CI [2.6–4.1]; aOR = 1.7, 95% CI [1.4–2.02]) and sometimes (aOR = 1.5, 95% CI [1.2–1.9]; aOR = 1.5, 95% CI [1.2–1.8]), respectively.

**Table 2:** Bullying victimization in both sexes

	Girls			Boys		
	Never	Sometimes	Often	Never	Sometimes	Often
	*n* (%)	*n* (%)	*n* (%)	*n* (%)	*n* (%)	*n* (%)
Age (years)						
<13	326 (40.6)	84 (33.9)	100 (27.6)	328 (35.5)	88 (42.7)	85 (37.9)
≥13	477 (59.4)	164 (66.1)	262 (72.4)	595 (64.5)	118 (57.3)	139 (62.1)
Region						
Kirovograd	376 (46.8)	138 (55.6)	129 (35.6)	458 (49.6)	102 (49.5)	100 (44.6)
Donetsk	427 (53.2)	110 (44.4)	233 (64.4)	465 (50.4)	104 (50.5)	124 (55.4)
Mother's employment status						
Employed	621 (77.3)	200 (80.6)	275 (75.9)	736 (79.7)	153 (74.3)	169 (75.4)
Unemployed	182 (22.7)	48 (19.4)	87 (24.03)	187 (20.3)	53 (25.7)	55 (24.6)
Father's employment status						
Employed	716 (89.3)	225 (90.7)	308 (85.1)	833 (90.2)	182 (88.3)	194 (86.6)
Unemployed	86 (10.7)	23 (9.3)	54 (14.9)	90 (9.8)	24 (11.7)	30 (13.4)
Family structure						
Biological parents	506 (63.01)	150 (60.5)	208 (57.5)	611 (66.2)	128 (62.1)	137 (61.2)
Others	297 (36.9)	98 (39.5)	154 (42.5)	312 (33.8)	78 (37.9)	87 (38.8)

	*M* (*SD*)	*M* (*SD*)	*M* (*SD*)	*M* (*SD*)	*M* (*SD*)	*M* (*SD*)
PTSD symptom scores	−0.1 (0.9)	0.4 (1.3)	0.6 (0.9)	−0.3 (0.7)	0.01 (1.1)	0.5 (1.1)
Depression symptom scores	−0.2 (0.8)	0.3 (1.2)	1.01 (1.1)	−0.4 (0.6)	0.2 (0.8)	0.6 (1.1)

**Table 3:** Association between bullying victimization in both sexes and with independent variables. Results of multinomial logistic regression

	Girls		Boys	
	Sometimes	Often	Sometimes	Often
	Multivariate analyses		Multivariate analyses	
	OR (95% CI)		OR (95% CI)	
Age				
<13 years	1.0	1.0	1.0	1.0
≥13	1.2 (0.8–1.6)	1.3 (0.9–1.8)	0.7 (0.5–0.9)*	0.8 (0.6–1.1)
Region				
Kirovograd	1.0	1.0	1.0	1.0
Donetsk	0.6 (0.4–0.8)***	1.2 (0.9–1.6)	1.02 (0.7–1.4)	1.3 (0.9–1.9)
Mother's employment status				
Employed	1.0	1.0	1.0	1.0
Unemployed	0.9 (0.7–1.4)	1.5 (1.1–2.1)*	1.4 (0.9–2.02)	1.3 (0.9–1.9)
Father's employment status				
Employed	1.0	1.0	1.0	1.0
Unemployed	0.8 (0.5–1.4)	1.11 (0.7–1.6)	1.1 (0.7–1.8)	0.9 (0.5–1.6)
Family structure				
Biological parents	1.0	1.0	1.0	1.0
Others	0.9 (0.7–1.3)	1.1 (0.8–1.4)	1.1 (0.8–1.5)	0.9 (0.7–1.3)
PTSD symptom scores^a^	1.7 (1.5–2.1)***	1.1 (0.9–1.3)	1.5 (1.2–1.8)***	1.7 (1.4–2.02)***
Depression symptom scores^a^	1.4 (1.2–1.6)***	2.9 (2.4–3.4)***	1.5 (1.2–1.9)***	3.3 (2.6–4.1)***

Multinomial logistic regression. Reference: Bully victimization never. CI, confidence interval; OR, odds ratio. *p<.05, ***p<.001

^a^ OR was calculated for one standard deviation change.

**Figure 3:**
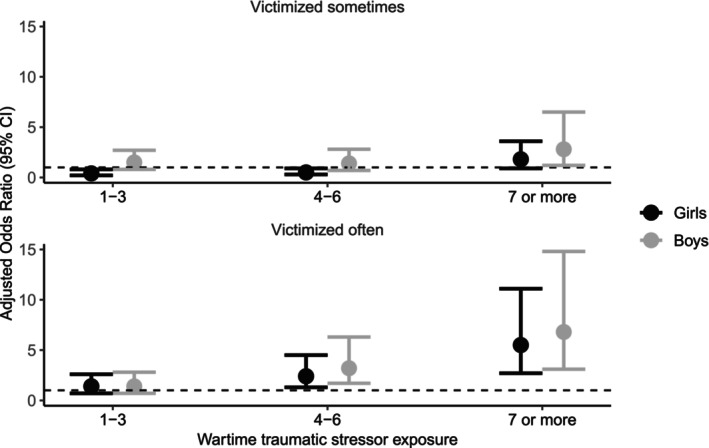
Association between level of wartime traumatic stressor exposure and bullying victimization: results from multinomial regression model

Figure 3 shows the association between the level of wartime traumatic stressor exposure and bullying victimization. The level of wartime traumatic stressor exposure was categorized into four groups: no exposure, 1–3 events, 4–6, and ≥7. In both regions and sexes, dose–response relationships were observed between wartime traumatic stressor exposure and bullying victimization. In the war‐affected region, the association with being victimized often was not significant if adolescent girls were exposed to one to three different wartime traumatic stressors (aOR = 1.4, 95% CI [0.7–2.6]) but the risk increased for those who had experienced four to six (aOR = 2.4, 95% CI [1.3–4.5]), see Table S2. The strongest association was for those who had been exposed to seven or more different trauma exposures (aOR = 5.5, 95% CI [2.7–11.1]). Similar results were reported in boys; no significant association was observed when exposed to one to three wartime traumatic stressor exposures (aOR = 1.4, 95% CI [0.7–2.8]), then increasing risk was reported when they were exposed to four to six (aOR = 3.2, 95% CI [1.7–6.3]) or seven or more different trauma exposures (aOR = 6.8, 95% CI [3.1–14.8]). The associations between specific wartime traumatic stressors and bullying victimization by sex are included; see Table S2. Both adolescent boys (aOR = 2.6, 95% CI [1.4–4.8]) and girls (aOR = 1.1, 95% CI [1.1–3.1]) who were victims of violence had increased risks for bullying victimization.

## Discussion

The present study showed that adolescent girls in the war‐affected region were victimized more often compared to those in the non‐war‐affected region. In contrast, no significant difference was found among boys. In both boys and girls, psychopathology symptoms were associated with bullying victimization. In the war‐affected region, a dose–response relationship between bullying victimization and the level of wartime traumatic stressor exposure was observed in both sexes. Importantly, while dose–response associations may imply causal relationships, other explanations are also possible. Related findings on dose–response relationships between traumatic exposure and children's behavioral and emotional problems, as well as posttraumatic symptoms, have previously been reported in different contexts (reviewed by Slone & Mann, 2016).

In the war‐affected region, girls reported higher rates of victimization compared to those in a less‐affected region, a disparity not observed among boys. These findings likely reflect underlying intricate and interconnected factors that influence experiences and reactions to wartime traumatic stressors. One such factor highlighted in previous literature relates to gender norms: It is possible that wartime traumatic stressors are perceived and experienced in relation to existing gender norms and, hence, their social significance (Kellezi & Reicher, 2014), which differs between males and females. Adolescent boys and girls may internalize social constructions regarding gender roles and inequalities (Cosma et al., 2022), legitimizing male aggression for control and enforcing power (Rosen & Nofziger, 2019), for example, in conflict situations as a means of enforcing conformity to established gender roles. It is also possible that chronic exposure to war or political violence may alter societal norms, fostering a culture of violence and thus influencing bullying behavior among peers (Timmer, Antonaccio, Botchkovar, Johnson, & Hughes, 2023).

The sex differences were also observed in the mean levels of depression and PTSD, which is in line with abundant international research showing generally higher mood and anxiety‐related psychopathology prevalence among women than men (Steel et al., 2014). In the present study, sex and psychopathology symptoms were further linked with bullying rates; for boys, depression and PTSD symptoms were associated with victimization. This was also the case for girls; however, to a somewhat lesser degree for PTSD symptoms. These results largely correspond with earlier research by Schwartz and Proctor (2000), who showed that community violence victimization was linked to bullying and peer rejection, mediated via emotion regulation. Difficulties in emotion regulation are a key element in both posttraumatic stress symptoms (Seligowski, Lee, Bardeen, & Orcutt, 2015) and depression (Joormann & Stanton, 2016).

Emotion regulation skills are also a central part of social functioning in that they facilitate adaptive and strategic self‐regulation in peer interactions (Shields, Cicchetti, & Ryan, 1994) and, hence, regulatory deficiencies may leave adolescents more vulnerable to bullying and peer rejection (Herd & Kim‐Spoon, 2021). Previous results from peaceful contexts have found bullying victimization to be associated with increased difficulties in emotion regulation (Valera‐Pozo, Flexas, Servera, Aguilar‐Mediavilla, & Adrover‐Roig, 2021). Such findings were also likely reflected in our results that indicated a relationship between cumulative wartime traumatic stressor exposure and bullying victimization. Multiple exposures to potentially traumatic events are associated with the increased likelihood of adolescents developing posttraumatic stress symptoms and depression (Suliman et al., 2009). Further, traumatic events involving deliberate interpersonal violence are particularly likely to result in PTSD (Santiago, Zazpe, Marti, Cuervo, & Martinez, 2013). Indeed, our results showed that violence toward oneself or a family member was specifically associated with bullying victimization. Posttraumatic symptoms, which are characterized by emotion dysregulation, may interfere with adolescents' social functioning with peers and leave them vulnerable to bullying. Furthermore, posttraumatic symptoms, such as heightened hypervigilance and an elevated perception of threat, can also contribute to adolescents being more susceptible to interpreting certain social interactions as harassment and thereby increasing the reports of experienced bullying. As per social learning theories, such heightened susceptibility may also be influenced by adolescents perceiving their parents as being fearful or traumatized.

In all, the interplay between social, emotional, psychological, and environmental factors in war settings associated with bullying is likely to be of a dynamic and complex nature and, therefore, warrants further examination.

The strengths of our study include the large‐scale representative sample and the possibility to compare between war‐affected and non‐war‐affected regions, which offers important clinical and theoretical implications. For example, both in schools and in clinical practice, it may be beneficial to introduce interventions strengthening bullying victims' emotion regulation skills. Teachers may be trained in preventive measures (e.g. discussion groups) on the role of war for interpersonal aggression between civilians.

However, this study has several limitations. First, the study is cross‐sectional and therefore causality cannot be inferred. Second, all variables are based on self‐reports, which may be vulnerable to response bias; however, self‐report is a norm in such studies. Third, the assessment of bullying victimization was based on only two items, and no information on cyberbullying was collected. Fourth, the psychometric properties of wartime traumatic stressor exposure have not been previously established.

## Conclusion

The present study aligns with the social learning theoretical perspective and contributes to the literature on how geo‐political contexts such as war may influence bullying behavior in adolescents. Adolescent girls were more likely to be victimized in the war context, proposedly through a complex interplay of social and individual factors, such as mental health symptoms. Bullying behavior in adolescents should be examined not only at the microsystem level within the individual (e.g. emotion regulation, psychopathology), family or school levels, but also within a broader macrosystem level, including factors such as development in politics and norms.

## Author contributions

A.S. and N.S. conceptualized the study design. O.S. carried out the data collection. S.S. analyzed the data. S.S. and M.W. drafted the manuscript. M.H. and S.H.‐Y.‐S. and all authors provided feedback.

## Funding information

The funding for this research was provided by the European Research Council (ERC) under the European Union's Horizon 2020 program (grant no. 101020767), and the Research Council of Finland (decision no: 345546) to AS. NS received funding from UNA (2016–2019) and SS from Juho Vainion Säätiö and Suomen Aivosäätiö. The funders played no role in the study or manuscript.

## Conflict of interest statement

The authors have declared that they have no competing or potential conflicts of interest.

## Ethics statement

Ethical approval was obtained from the ethical committee for the medical research ethics of Donetsk National Medical University, Ukraine on 11 May 2016, number 16. Informed consent was obtained from the parents and adolescents.

## Supporting information


**Table S1.** Comparison of bullying victimization in two regions and gender.
**Table S2.** Association between war trauma exposure and bullying victimization in the war‐affected Donetsk.
**Table S3.** Raw estimates of the regression model.

## Data Availability

The data that support the findings of this study are available on request from the corresponding author. The data are not publicly available due to privacy or ethical restrictions.
