# Supplementary material for: Bullying victimization among adolescents during the early phase of war in Ukraine – A comparative cross‐sectional study in 2016–2017
Source: Child Adolesc Ment Health. 2025 Mar 18;30(3):247–55. doi: 10.1111/camh.12770 (PMC12351195; doi:10.1111/camh.12770)
Supplement: Supplementary file 1 — Table S1. Comparison of bullying victimization in two regions and gender. Table S2. Association between war trauma exposure and bullying victimization in the war‐affected Donetsk. Table S3. Raw estimates of the regression model. [file CAMH-30-247-s001.docx]

**Supporting Information**

**Table S1. Comparison of bullying victimization in two regions and gender**

|  | **Total** | **Donetsk region (war-affected region)**  ***(n=1463)*** | | | **Kirovograd region (non-war region)**  ***(n=1303)*** | | | **Boys in war-affected region vs boys in non-war region** | **Girls in war-affected region vs girls in non-war region** |
| --- | --- | --- | --- | --- | --- | --- | --- | --- | --- |
|  |  | **Boys** | **Girls** | **OR (95% CI)^a^** | **Boys** | **Girls** | **OR (95% CI) ^a^** | **OR (95% CI) ^a^** | **OR (95% CI) ^a^** |
| **Victim of bullying** | ***n (%)*** | ***n***  ***(%)*** | ***n***  ***(%)*** |  | ***n***  ***(%)*** | ***n***  ***(%)*** |  |  |  |
| Never | 1726 (62.4) | 465 (52.1) | 427 (47.9) | 1.0 | 458 (54.9) | 376 (45.1) |  | 1.0 | 1.0 |
| Sometimes | 454 (16.4) | 104 (48.6) | 110 (51.4) | 1.5 (0.9-1.6) | 102 (42.5) | 138 (57.5) | 1.7 (1.2-2.2)*** | 0.9 (0.7-1.3) | 0.7 (0.5-0.9)* |
| Often | 586 (21.2) | 124 (34.7) | 233 (65.3) | 2.04 (1.6-2.6)*** | 100 (43.7) | 129 (56.3) | 1.6 (1.2-2.1)** | 1.2 (0.9-1.6) | 1.5 (1.2-1.9)** |

CI = Confidence interval; OR = Odds ratio.

Reference: Victim of bullying (never), boys and non-war region.

Multinomial logistic regression. Reference: Bullying victimization never.

**^a^**Adjusted for age and father employment status.

*p<0.05, **p<0.01, ***p<0.001

**Table S2. Association between war trauma exposure and bullying victimization in the war-affected Donetsk region**

|  | **Girls** | | | | **Boys** | | | |
| --- | --- | --- | --- | --- | --- | --- | --- | --- |
|  | **Bullied sometimes** | | **Bullied often** | | **Bullied sometimes** | | **Bullied often** | |
| **War trauma exposure** | ***n (%)*** | **OR (95% CI)** | ***n (%)*** | **OR (95% CI)** | ***n (%)*** | **OR^a^ (95% CI)** | ***n (%)*** | **OR^a^ (95% CI)** |
| No exposure | 22 (23.7) | 1.0 | 15 (16.1) | 1.0 | 16 (12.2) | 1.0 | 13 (9.9) | 1.0 |
| 1-3 | 37 (11.3) | 0.4 (0.2-0.8) | 79 (24.2) | 1.4 (0.7-2.6) | 49 (16.0) | 1.5 (0.8-2.7) | 39 (12.7) | 1.4 (0.7-2.8) |
| 4-6 | 24 (10.6) | 0.5 (0.3-0.9) | 80 (35.2) | 2.4 (1.3-4.5)** | 26 (13.8) | 1.4 (0.7-2.8) | 47 (24.9) | 3.2 (1.7-6.3)*** |
| ≥7 | 27 (22.0) | 1.8 (0.9-3.6) | 59 (48.0) | 5.5 (2.7-11.1)*** | 13 (19.4) | 2.8 (1.2-6.5)* | 25 (37.3) | 6.8 (3.1-14.8)*** |
| **Direct violence** |  |  |  |  |  |  |  |  |
| Victim of violence or used as a human shield | 34 (27.1) | 3.6 (2.01-6.4)*** | 52 (41.3) | 1.8 (1.1-3.01)* | 11 (14.1) | 1.1 (0.5-2.5) | 31 (29.7) | 2.6 (1.4-4.8)** |
| Threatened by armed forces or military events^c^ | 39 (17.6) | 1.3 (0.8-2.1) | 71 (32.1) | 0.9 (0.7-1.4) | 30 (16.3) | 0.9 (0.6-1.6) | 35 (19.1) | 0.7 (0.4-1.2) |
| Witnessed homes destroyed, broken windows^d^ | 51 (16.7) | 1.4 (0.8-2.4) | 110 (35.9) | 1.4 (0.9-2.1) | 38 (16.1) | 1.5 (0.9-2.7) | 48 (20.2) | 0.9 (0.6-1.7) |
| Witnessed civilians killed or injured or intimidated^e^ | 45 (17.4) | 1.1 (0.6-1.9) | 94 (36.3) | 1.2 (0.8-1.8) | 23 (12.6) | 0.6 (0.3-1.1) | 43 (23.5) | 1.3 (0.8-2.2) |
| Witnessed attacks by armed forces, artillery fire or explosions | 69 (13.8) | 0.6 (0.4-1.1) | 159 (31.9) | 0.9 (0.6-1.3) | 54 (14.1) | 0.8 (0.5-1.4) | 72 (18.8) | 0.8 (0.5-1.3) |
| Property/housing looted, confiscated, destroyed, or lacked shelter^f^ | 7 (13.2) | 0.9 (0.4-2.4) | 20 (37.7) | 1.2 (0.6-2.2) | 13 (23.2) | 2.2 (0.9-4.8) | 19 (33.9) | 1.5 (0.7-3.2) |
| **Non-violent** |  |  |  |  |  |  |  |  |
| Forced to leave hometown | 25 (11.1) | 0.7 (0.4-1.2) | 96 (42.7) | 1.3 (0.9-2.1) | 33 (17.9) | 1.1 (0.6-2.01) | 51 (27.7) | 1.1 (0.6-1.9) |
| Lost social support networks | 37 (15.9) | 1.6 (0.9-2.8) | 100 (42.9) | 1.7 (1.1-2.6)* | 29 (18.8) | 1.2 (0.6-2.4) | 44 (28.6) | 1.2 (0.7-2.2) |
| Forced separation from parents or family members^g^ | 19 (18.6) | 1.7 (0.9-3.3) | 47 (46.1) | 1.6 (0.9-2.7) | 13 (17.1) | 1.2 (0.6-2.7) | 30 (39.5) | 1.9 (0.9-3.7) |
| Difficult to adapt in new location | 27 (14.5) | 1.3 (0.7-2.4) | 82 (44.1) | 1.6 (0.9-2.5) | 29 (18.8) | 1.3 (0.7-2.6) | 48 (31.2) | 1.7 (0.9-3.1) |
| Seeking safer places/hiding in basements/air raid shelter | 44 (17.9) | 1.4 (0.8-2.3) | 83 (34.1) | 1.1 (0.7-1.5) | 18 (12.3) | 0.7 (0.4-1.3) | 33 (22.6) | 1.02 (0.6-1.8) |
| Frequent or long hours roadblocks due to military checkpoints^h^ | 34 (11.9) | 0.5 (0.3-0.9)* | 99 (34.9) | 0.7 (0.5-1.1) | 34 (16.7) | 1.2 (0.7-2.04) | 54 (26.6) | 1.5 (0.9-2.4) |

CI = Confidence Interval; OR = Odds Ratio.

Multinomial logistic regression model. Reference: Bully victimization never.

^a^Ajdusted for age and father employment.

*p<0.05, **p<0.01, ***p<0.001.

**Table S3. Raw estimates of the regression model.**

| **Model Fitting Information** | | | | | |
| --- | --- | --- | --- | --- | --- |
| Sex | Model | Model Fitting Criteria | Likelihood Ratio Tests | | |
|  |  | -2 Log Likelihood | Chi-Square | df | Sig. |
| Girl | Intercept Only | 2503,671 |  |  |  |
|  | Final | 2157,861 | 345,811 | 14 | <.001 |
| Boy | Intercept Only | 1943,672 |  |  |  |
|  | Final | 1653,380 | 290,292 | 14 | <.001 |

| **Case Processing Summary** | | | | |
| --- | --- | --- | --- | --- |
| Sex | | | N | Marginal Percentage |
| Girl | Bully victimization | Not at all | 797 | 56.7% |
|  |  | Sometimes | 247 | 17.6% |
|  |  | Often | 362 | 25.7% |
|  | Age | Equal or more than 13 | 899 | 63.9% |
|  |  | <13 | 507 | 36.1% |
|  | Region | Donetsk | 766 | 54.5% |
|  |  | Kirovograd | 640 | 45.5% |
|  | Father's employment status | Unemployed | 163 | 11.6% |
|  |  | Employed | 1243 | 88.4% |
|  | Mother's employment status | Unemployed | 317 | 22.5% |
|  |  | Employed | 1089 | 77.5% |
|  | Family structure | Others | 544 | 38.7% |
|  |  | Biological parents | 862 | 61.3% |
|  | Valid | | 1406 | 100.0% |
|  | Missing | | 7 |  |
|  | Total | | 1413 |  |
|  | Subpopulation | | 1021^a^ |  |
| Boy | Bully victimization | Not at all | 917 | 68.2% |
|  |  | Sometimes | 206 | 15.3% |
|  |  | Often | 222 | 16.5% |
|  | Age | Equal or more than 13 | 849 | 63.1% |
|  |  | <13 | 496 | 36.9% |
|  | Region | Donetsk | 689 | 51.2% |
|  |  | Kirovograd | 656 | 48.8% |
|  | Father's employment status | Unemployed | 144 | 10.7% |
|  |  | Employed | 1201 | 89.3% |
|  | Mother's employment status | Unemployed | 294 | 21.9% |
|  |  | Employed | 1051 | 78.1% |
|  | Family structure | Others | 473 | 35.2% |
|  |  | Biological parents | 872 | 64.8% |
|  | Valid | | 1345 | 100.0% |
|  | Missing | | 8 |  |
|  | Total | | 1353 |  |
|  | Subpopulation | | 798^b^ |  |
| a. The dependent variable has only one value observed in 920 (90.1%) subpopulations. | | | | |
| b. The dependent variable has only one value observed in 686 (86.0%) subpopulations. | | | | |

| **Pseudo R-Square** | | |
| --- | --- | --- |
| Girl | Cox and Snell | .218 |
|  | Nagelkerke | .254 |
|  | McFadden | .126 |
| Boy | Cox and Snell | .194 |
|  | Nagelkerke | .238 |
|  | McFadden | .128 |

| **Likelihood Ratio Tests** | | | | | |
| --- | --- | --- | --- | --- | --- |
| Sex | Effect | Model Fitting Criteria | Likelihood Ratio Tests | | |
|  |  | -2 Log Likelihood of Reduced Model | Chi-Square | df | Sig. |
| Girl | Intercept | 2157.861^a^ | .000 | 0 | . |
|  | Age | 2161.123 | 3.262 | 2 | .196 |
|  | Region | 2174.151 | 16.290 | 2 | <.001 |
|  | Father's employment status | 2158.714 | .853 | 2 | .653 |
|  | Mother's employment status | 2164.025 | 6.164 | 2 | .046 |
|  | Family structure | 2158.084 | .223 | 2 | .894 |
|  | Depression | 2350.671 | 192.810 | 2 | <.001 |
|  | PTSD | 2171.901 | 14.040 | 2 | <.001 |
| Boy | Intercept | 1653.380^a^ | .000 | 0 | . |
|  | Age | 1658.733 | 5.353 | 2 | .069 |
|  | Region | 1655.965 | 2.586 | 2 | .275 |
|  | Father's employment status | 1653.539 | .159 | 2 | .923 |
|  | Mother's employment status | 1657.612 | 4.232 | 2 | .121 |
|  | Family structure | 1654.122 | .742 | 2 | .690 |
|  | Depression | 1788.477 | 135.097 | 2 | <.001 |
|  | PTSD | 1687.304 | 33.925 | 2 | <.001 |
| The chi-square statistic is the difference in -2 log-likelihoods between the final model and a reduced model. The reduced model is formed by omitting an effect from the final model. The null hypothesis is that all parameters of that effect are 0. | | | | | |
| a. This reduced model is equivalent to the final model because omitting the effect does not increase the degrees of freedom. | | | | | |

| **Parameter Estimates** | | | | | | | | | | | |
| --- | --- | --- | --- | --- | --- | --- | --- | --- | --- | --- | --- |
| Sex | Bully victimization^a.c^ | | B | Std. Error | Wald | df | Sig. | Exp(B) | 95% Confidence Interval for Exp(B) | |  |
|  |  |  |  |  |  |  |  |  | Lower Bound | Upper Bound |  |
| Girl | Sometimes | Intercept | -1.018 | .160 | 40.544 | 1 | <.001 |  |  |  |  |
|  |  | [Age=1.00] | .147 | .159 | .860 | 1 | .354 | 1.158 | .849 | 1.580 |  |
|  |  | [Age=2.00] | 0^b^ | . | . | 0 | . | . | . | . |  |
|  |  | [City=1.00] | -.524 | .155 | 11.383 | 1 | <.001 | .592 | .437 | .803 |  |
|  |  | [City=2.00] | 0^b^ | . | . | 0 | . | . | . | . |  |
|  |  | [Father's employment status=1.00] | -.191 | .261 | .534 | 1 | .465 | .826 | .495 | 1.379 |  |
|  |  | [Father's employment status=2.00] | 0^b^ | . | . | 0 | . | . | . | . |  |
|  |  | [Mother's employment status=1.00] | -.055 | .188 | .086 | 1 | .769 | .946 | .654 | 1.368 |  |
|  |  | [Mother's employment status=2.00] | 0^b^ | . | . | 0 | . | . | . | . |  |
|  |  | [Family structure=1.00] | -.028 | .156 | .032 | 1 | .859 | .973 | .716 | 1.321 |  |
|  |  | [Family structure=2.00] | 0^b^ | . | . | 0 | . | . | . | . |  |
|  |  | Depression | .556 | .092 | 36.614 | 1 | <.001 | 1.744 | 1.456 | 2.088 |  |
|  |  | PTSD | .319 | .084 | 14.302 | 1 | <.001 | 1.376 | 1.166 | 1.623 |  |
|  | Often | Intercept | -1.480 | .168 | 77.277 | 1 | <.001 |  |  |  |  |
|  |  | [Age=1.00] | .269 | .154 | 3.051 | 1 | .081 | 1.308 | .968 | 1.769 |  |
|  |  | [Age=2.00] | 0^b^ | . | . | 0 | . | . | . | . |  |
|  |  | [City=1.00] | .152 | .149 | 1.036 | 1 | .309 | 1.164 | .869 | 1.559 |  |
|  |  | [City=2.00] | 0^b^ | . | . | 0 | . | . | . | . |  |
|  |  | [Father's employment status=1.00] | .058 | .220 | .069 | 1 | .793 | 1.060 | .688 | 1.632 |  |
|  |  | [Father's employment status=2.00] | 0^b^ | . | . | 0 | . | . | . | . |  |
|  |  | [Mother's employment status=1.00] | .386 | .169 | 5.211 | 1 | .022 | 1.472 | 1.056 | 2.051 |  |
|  |  | [Mother's employment status=2.00] | 0^b^ | . | . | 0 | . | . | . | . |  |
|  |  | [Family structure=1.00] | .052 | .147 | .125 | 1 | .724 | 1.053 | .789 | 1.406 |  |
|  |  | [Family structure=2.00] | 0^b^ | . | . | 0 | . | . | . | . |  |
|  |  | Depression | 1.048 | .085 | 153.157 | 1 | <.001 | 2.853 | 2.416 | 3.368 |  |
|  |  | PTSD | .134 | .082 | 2.702 | 1 | .100 | 1.144 | .974 | 1.343 |  |
| Boy | Sometimes | Intercept | -1.197 | .167 | 51.607 | 1 | <.001 |  |  |  |  |
|  |  | [Age=1.00] | -.351 | .160 | 4.821 | 1 | .028 | .704 | .515 | .963 |  |
|  |  | [Age=2.00] | 0^b^ | . | . | 0 | . | . | . | . |  |
|  |  | [City=1.00] | .017 | .162 | .011 | 1 | .916 | 1.017 | .740 | 1.398 |  |
|  |  | [City=2.00] | 0^b^ | . | . | 0 | . | . | . | . |  |
|  |  | [Father's employment status=1.00] | .059 | .260 | .052 | 1 | .820 | 1.061 | .637 | 1.767 |  |
|  |  | [Father's employment status=2.00] | 0^b^ | . | . | 0 | . | . | . | . |  |
|  |  | [Mother's employment status=1.00] | .340 | .184 | 3.413 | 1 | .065 | 1.405 | .979 | 2.017 |  |
|  |  | [Mother's employment status=2.00] | 0^b^ | . | . | 0 | . | . | . | . |  |
|  |  | [Family structure=1.00] | .105 | .165 | .407 | 1 | .524 | 1.111 | .804 | 1.535 |  |
|  |  | [Family structure=2.00] | 0^b^ | . | . | 0 | . | . | . | . |  |
|  |  | Depression | .429 | .128 | 11.274 | 1 | <.001 | 1.536 | 1.196 | 1.974 |  |
|  |  | PTSD | .381 | .095 | 15.954 | 1 | <.001 | 1.463 | 1.214 | 1.763 |  |
|  | Often | Intercept | -1.375 | .185 | 55.312 | 1 | <.001 |  |  |  |  |
|  |  | [Age=1.00] | -.220 | .177 | 1.543 | 1 | .214 | .802 | .567 | 1.136 |  |
|  |  | [Age=2.00] | 0^b^ | . | . | 0 | . | . | . | . |  |
|  |  | [City=1.00] | .282 | .178 | 2.509 | 1 | .113 | 1.325 | .935 | 1.877 |  |
|  |  | [City=2.00] | 0^b^ | . | . | 0 | . | . | . | . |  |
|  |  | [Father's employment status=1.00] | -.074 | .290 | .066 | 1 | .798 | .928 | .526 | 1.639 |  |
|  |  | [Father's employment status=2.00] | 0^b^ | . | . | 0 | . | . | . | . |  |
|  |  | [Mother's employment status=1.00] | .282 | .205 | 1.904 | 1 | .168 | 1.326 | .888 | 1.981 |  |
|  |  | [Mother's employment status=2.00] | 0^b^ | . | . | 0 | . | . | . | . |  |
|  |  | [Family structure=1.00] | -.076 | .184 | .173 | 1 | .678 | .926 | .646 | 1.328 |  |
|  |  | [Family structure=2.00] | 0^b^ | . | . | 0 | . | . | . | . |  |
|  |  | Depression | 1.194 | .115 | 107.006 | 1 | <.001 | 3.300 | 2.632 | 4.138 |  |
|  |  | PTSD | .508 | .095 | 28.813 | 1 | <.001 | 1.662 | 1.381 | 2.002 |  |
| a. The reference category is: Not at all for split file Sex = Girl. | | | | | | | | | | | |
| b. This parameter is set to zero because it is redundant. | | | | | | | | | | | |
| c. The reference category is: Not at all for split file Sex = Boy. | | | | | | | | | | | |

| **Classification** | | | | | |
| --- | --- | --- | --- | --- | --- |
| Sex | Observed | Predicted | | | |
|  |  | Not at all | Sometimes | Often | Percent Correct |
| Girl | Not at all | 746 | 1 | 50 | 93.6% |
|  | Sometimes | 191 | 3 | 53 | 1.2% |
|  | Often | 186 | 1 | 175 | 48.3% |
|  | Overall Percentage | 79.9% | 0.4% | 19.8% | 65.7% |
| Boy | Not at all | 892 | 0 | 25 | 97.3% |
|  | Sometimes | 184 | 0 | 22 | 0.0% |
|  | Often | 142 | 0 | 80 | 36.0% |
|  | Overall Percentage | 90.6% | 0.0% | 9.4% | 72.3% |
